# Comment on Article “Risk Models for Developing Pancreatic Fistula After Pancreatoduodenectomy: Validation in a Nationwide Prospective Cohort”

**DOI:** 10.1097/AS9.0000000000000392

**Published:** 2024-02-19

**Authors:** Yanfei Yang, Ziqiang Du, Rui Du

**Affiliations:** * From the Department of General Surgery, The First Affiliated Hospital of Dalian Medical University, Dalian, China.

We have recently been very interested and discussed in depth an article entitled “Risk Models for Developing Pancreatic Fistula After Pancreatoduodenectomy: Validation in a Nationwide Prospective Cohort.”^1^ The performance of the published fistula risk model was evaluated by external validation of the Dutch medical database. The published risk factors were included and the relevant clinical data were statistically analyzed. Finally, the predictive performance of the model was evaluated by area under receiver opreating characteristic and calibration, and finally, the prediction model of postoperative pancreatic fistula (POPF) was constructed.^1^

We are very grateful for the research done by the author, but there are still some deficiencies that we need to discuss together.

First of all, according to the latest relevant research, computed tomography visceral fat area (*P* = 0.014), the pancreatic spleen signal ratio on T1 fat-suppressed magnetic resonance imaging sequences (*P* < 0.001), and computed tomography main pancreatic duct diameter (*P* = 0.001) are identified as independent prognostic factors.^2^ If these 2 imaging risk factors can be included in the model discussion, the construction dimension of POPF’s model will be improved accordingly, and its scientificity will also be improved.^3^ Second, according to relevant reports, the ratio of drainage fluid to serum amylase concentration (area under curve: 0.77, sensitivity: 77.7%, specificity: 73.3%, and accuracy: 73.6%) is an independent risk factor for POPF, and this risk factor deserves to be included in the POPF prediction model for modeling, so as to improve the comprehensiveness of the model.^4^ Finally, only receiver operating charactiristic curve and calibration graph are applied to the verified model for prediction and evaluation.^3^ If decision curve analysis is added, the prediction and evaluation effect will be more accurate and true. Finally, we would like to thank the authors again for their contribution. This study provides a further reference to clinic related-postoperative pancreatic fistula prediction studies from a new classification basis, and its significance deserves further investigation.

## References

[1] Schouten TJ Henry AC Smits FJ; Dutch Pancreatic Cancer Group. Risk models for developing pancreatic fistula after pancreatoduodenectomy: validation in a nationwide prospective cohort. Ann Surg. 2023;278:1001–1008. 36804843 10.1097/SLA.0000000000005824

[2] Zou J Xue X Qin L. Development of a nomogram to predict clinically relevant postoperative pancreatic fistula after pancreaticoduodenectomy on the basis of visceral fat area and magnetic resonance imaging. Ann Surg Oncol. 2023;30:7712–7719. 37530992 10.1245/s10434-023-13943-0

[3] Bonsdorff A Sallinen V. Prediction of postoperative pancreatic fistula and pancreatitis after pancreatoduodenectomy or distal pancreatectomy: a review. Scand J Surg. 2023;112:126–134. 37083016 10.1177/14574969231167781

[4] Fukada M Murase K Higashi T. Drain fluid and serum amylase concentration ratio is the most reliable indicator for predicting postoperative pancreatic fistula after distal pancreatectomy. BMC Surg. 2023;23:87. 37046241 10.1186/s12893-023-01980-1 PMC10091553

